# Physio-biochemical and molecular mechanism underlying the enhanced heavy metal tolerance in highland barley seedlings pre-treated with low-dose gamma irradiation

**DOI:** 10.1038/s41598-017-14601-8

**Published:** 2017-10-27

**Authors:** Xiaojie Wang, Ruonan Ma, Dongjie Cui, Qing Cao, Zhe Shan, Zhen Jiao

**Affiliations:** Henan Key Laboratory of Ion- beam Bioengineering, Zhengzhou University, Zhengzhou, 450052 China

## Abstract

Heavy metal pollution, as a consequence of rapid industrialization and urbanization, poses a threat to highland barley grown in Tibet. This study investigates the effect of different doses of gamma irradiation (50–300 Gy) on the physio-biochemical and molecular mechanism of highland barley under heavy metal stress. Growth data showed that 50-Gy gamma irradiation had the maximal beneficial effects on the highland barley seedlings under lead/cadmium stress. The results of oxidative parameters demonstrated that 50-Gy gamma-irradiated seedlings had lower hydrogen peroxide and malondialdehyde contents under lead/cadmium stress compared to non-irradiated seedlings. Moreover, the activities of antioxidant enzyme and proline levels in 50-Gy gamma-irradiated seedlings were drastically higher than those in non-irradiated seedlings under lead/cadmium stress. Additionally, transmission electron microscopy results revealed that the 50-Gy gamma-irradiated seedlings exhibited improved chloroplasts ultrastructure compared with non-irradiated seedlings exposed to lead/cadmium stress. Notably, transcriptional expression analysis showed that 50-Gy gamma irradiation could significantly affect the expression of genes related to heavy metal transport and abscisic acid metabolism under lead/cadmium stress. Collectively, these results provide insights into the physio-biochemical and molecular mechanisms of low-dose-gamma-irradiation-enhanced heavy metal tolerance in highland barley seedlings, thus proposing gamma irradiation as a potential technology to mitigate heavy metal toxicity in crops.

## Introduction

Heavy metal pollution is emerging as a serious environmental and health issue. Heavy metals accumulating in crops are transferred to the human food chain, causing a serious threat to human health, reduction in food and feed quality as well as economic losses^1–5^. The Tibetan Plateau within the Tibet Autonomous Region (T.A.R., China) covers an area of 1.22 million km^2^, and has an average elevation of more than 4000 m above sea level. Within this region, large deposits of various sorts of mineral resources have been discovered since the early 1950s^6^. The Tibetan Plateau is traditionally considered to be a pristine environment. However, in recent years, its ecological environment has been subjected to heavy metal accumulation as a result of rapid economic development, urbanization, industrialization and the completion of the Qinghai–Tibet railway^7–10^. Among these factors, industrial scale metal mining operations are considered to contribute most to the heavy metal pollution in the Tibetan Plateau due to the lack of adequate management and planning, as well as poor operating practices and waste management^11^. Consequently, heavy metal pollution poses a potential threat to the croplands of the Tibetan Plateau.

Highland barley, also called naked barley, is widely cultivated in the Tibetan Plateau and considered as one of the most economical and nutritious foods for the Tibetan population, which can be made into a variety of conventional foods, such as fried noodles (i.e. zanba), wine, and health food rich in high beta-glucan^12–14^. However, cultivation of highland barley in heavy metal polluted lands results in yield loss, reduced seed quality, and heavy metal toxicity in humans. Therefore, there is an urgent need to improve the heavy metal tolerance in highland barely and understand the overall mechanisms regulating tolerance.

Lead (Pb) and cadmium (Cd), as the non-essential heavy metals, are persistent environmental contaminants, causing serious toxicity to all living organisms^15^. Pb and Cd are also water soluble heavy metals, which can be quickly taken up by plant roots, and transported to the aerial parts where their accumulation significantly impede vital cellular processes, commonly leading to chlorosis, necrosis, epinasty, stunted growth, cell death, disturbance in mineral homeostasis and reduced biomass^16,17^. At the subcellular level, excessive Pb and Cd can inactivate the biomolecules by either blocking essential functional groups or displacing essential metal ions; they can also induce oxidative stress by generating reactive oxygen species (ROS)^17^. Plants have developed several strategies for heavy metal detoxification, which includes binding of heavy metals to cell wall and extracellular exudates, chelation of metal ions in cytosol, compartmentation of metals in vacuoles or other subcellular structures, repair of damaged biomolecules and pumping metal ions from cytosol to the apoplast via subcellular heavy metal transporters, such as *HvHMT1*, *HvHMT2*, *HvHMT3* and *HvHMT4* in barley^18^. Additionally, it is well known that plant hormone abscisic acid (ABA) plays great roles in the adaptive responses to environmental stresses as well as normal growth and development for higher plants^19^. Recent data suggested that the gene expression of *HvZEP1*, *HvZEP2*, *HvNCED1*, *HvNCED2*, *HvAO1*, *HvAO2* and *HvAO3* were positively correlated with ABA level. While, *HvABA8*′*OH-1*, *HvABA8*′*OH-2*, *HvABA8*′*OH-3*, *HvBG1* and *HvBG2* were ABA catabolic genes^20^. Furthermore, to combat heavy metal-induced oxidative stress, plant cells are well equipped with intrinsic antioxidant capacity comprising enzymatic components, such as superoxide dismutase (SOD), catalase (CAT), and peroxidase (POD), as well as non-enzymatic components, such as glutathione (GSH) and proline^21^.

In the past few decades, gamma irradiation has been widely applied in mutation breeding and plant improvement due to its penetration power^22–25^. Gamma irradiation can affect plant growth and development by inducing cytological, biochemical, physiological and morphological changes in cells and tissues^26^. The relative low dose of gamma irradiation have positive effects on plants, such as increasing cell proliferation, germination, cell growth, enzyme activity and crop yields, while high-dose gamma irradiations cause inhibitory effects, which was named radiation hormesis^27^. More recently, the investigations of low-dose gamma irradiation on the plant resistance to abiotic stress have attracted much attention because of the continuous deterioration of environments. Several researchers have found that the gamma irradiations at low doses can improve the tolerance of plants to various stresses, including cold, drought, water, heat, salt, and heavy metal^28–31^. However, there is still little information about the effects of gamma irradiation on heavy metal tolerance in highland barley seedlings.

Hence, this study was carried out to investigate the effects of gamma irradiation on the physio-biochemical and molecular mechanism of highland barley seedlings to heavy metal stress. The dry seeds of highland barley were exposed to a Cobalt-60 (^60^Co) gamma source at doses ranging from 0 to 300- Gy before being subjected to 500 μM Pb(NO_3_)_2_ or 75 μM CdCl_2_. Physio-biochemical responses to Pb/Cd stress were estimated by measurement of phenotype, growth parameters (plant height, root length, leaf area and fresh weight), oxidative stress parameters (malondialdehyde (MDA) and hydrogen peroxide (H_2_O_2_)), antioxidants (SOD, POD and CAT) and proline in irradiated (50–300 Gy) and non-irradiated (0- Gy) highland barley seedlings. Moreover, the ultrastructural change in chloroplast morphology was detected by transmission electron microscopy (TEM). Furthermore, with respect to molecular mechanism, altered expression levels of key genes related to heavy metal transport (*HvHMT1*, *HvHMT2*, *HvHMT3* and *HvHMT4*), ABA biosynthesis (*HvZEP1*, *HvZEP2*, *HvNCED1*, *HvNCED2*, *HvAO1*, *HvAO2* and *HvAO3*) and ABA catabolism (*HvABA8*′*OH-1*, *HvABA8*′*OH-2*, *HvABA8*′*OH-3*, *HvBG1* and *HvBG2*) were analyzed by real-time RT-PCR.

## Results

### The overall experimental procedure

**Figure 1:**
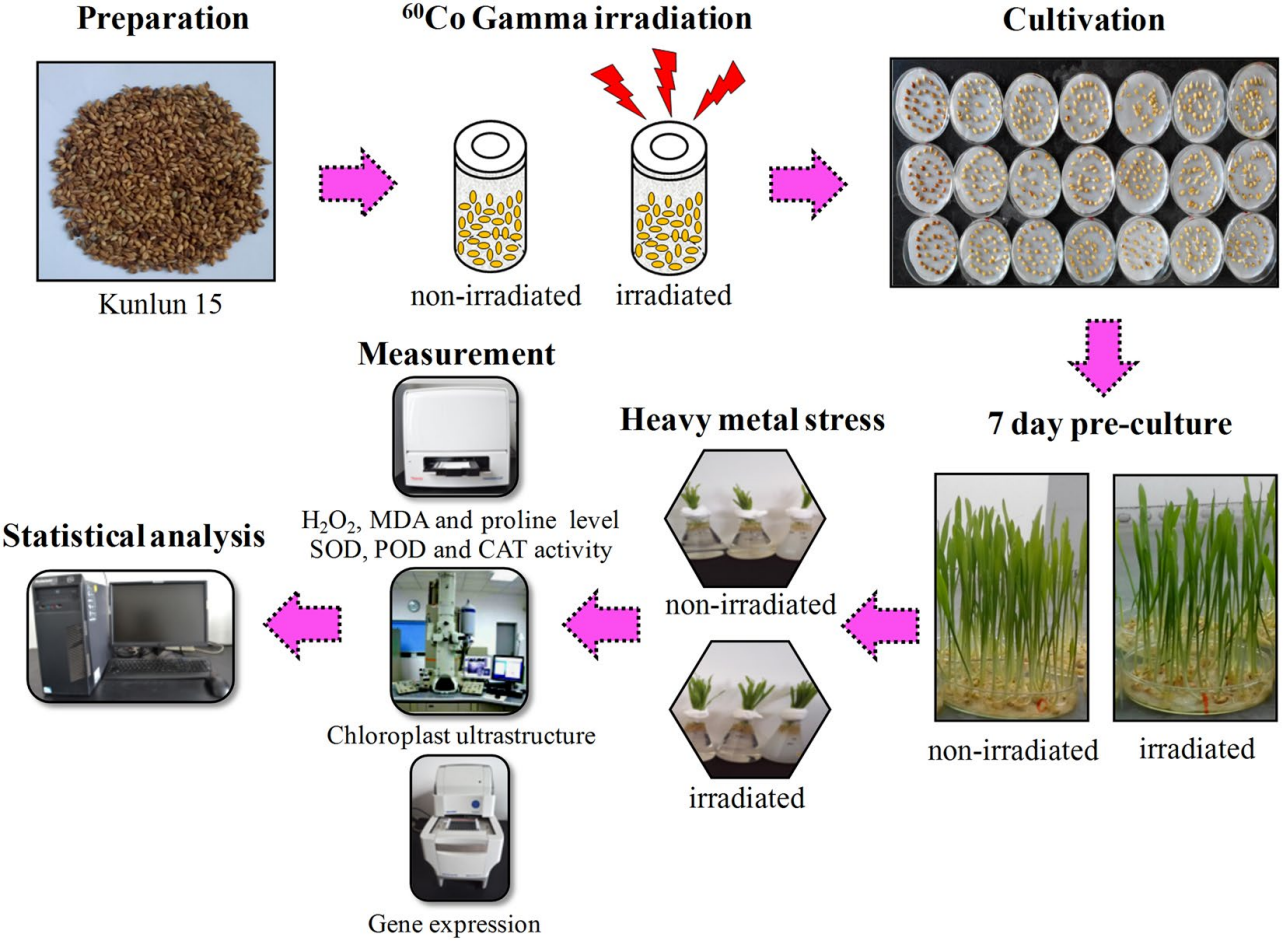
A schematic diagram of the experimental procedure in this study, including seed preparation, ^60^Co gamma irradiation, seed cultivation, heavy metal treatment, various parameters measurement and data analysis.

The experimental protocol for heavy metal treatment of highland barley seedlings pre-treated with different doses of gamma irradiation and the following measurements are shown in Fig. 1. Briefly, the uniform seeds of highland barley cultivar ‘Kunlun15′ were randomly divided into two groups: non-irradiated seeds (with 0-Gy ^60^Co gamma irradiation treatment) and irradiated seeds (with 50, 100, 150, 200, 250 and 300-Gy ^60^Co gamma irradiation treatment, respectively). The seeds were germinated at 25 °C in the dark for 72 h. The germinated seeds were transferred into a plant growth chamber at 23 °C with a 16-h-light/8-h-dark cycle. For Pb/Cd stress, 7-day-old non-irradiated and irradiated highland barley seedlings were submerged in Hoagland’s solution containing 500 µM Pb(NO_3_)_2_ or 75 µM CdCl_2_ for 48 h. Then, the H_2_O_2_, MDA and proline contents as well as SOD, POD and CAT activities of highland barley seedlings were measured by a rapid scanning ultraviolet-visible spectrometer. Chloroplast ultra-structure was detected by TEM and gene expression was analyzed by real-time RT-PCR.

### Effect of gamma irradiation on highland barley phenotype and growth under Pb/Cd stress

**Figure 2:**
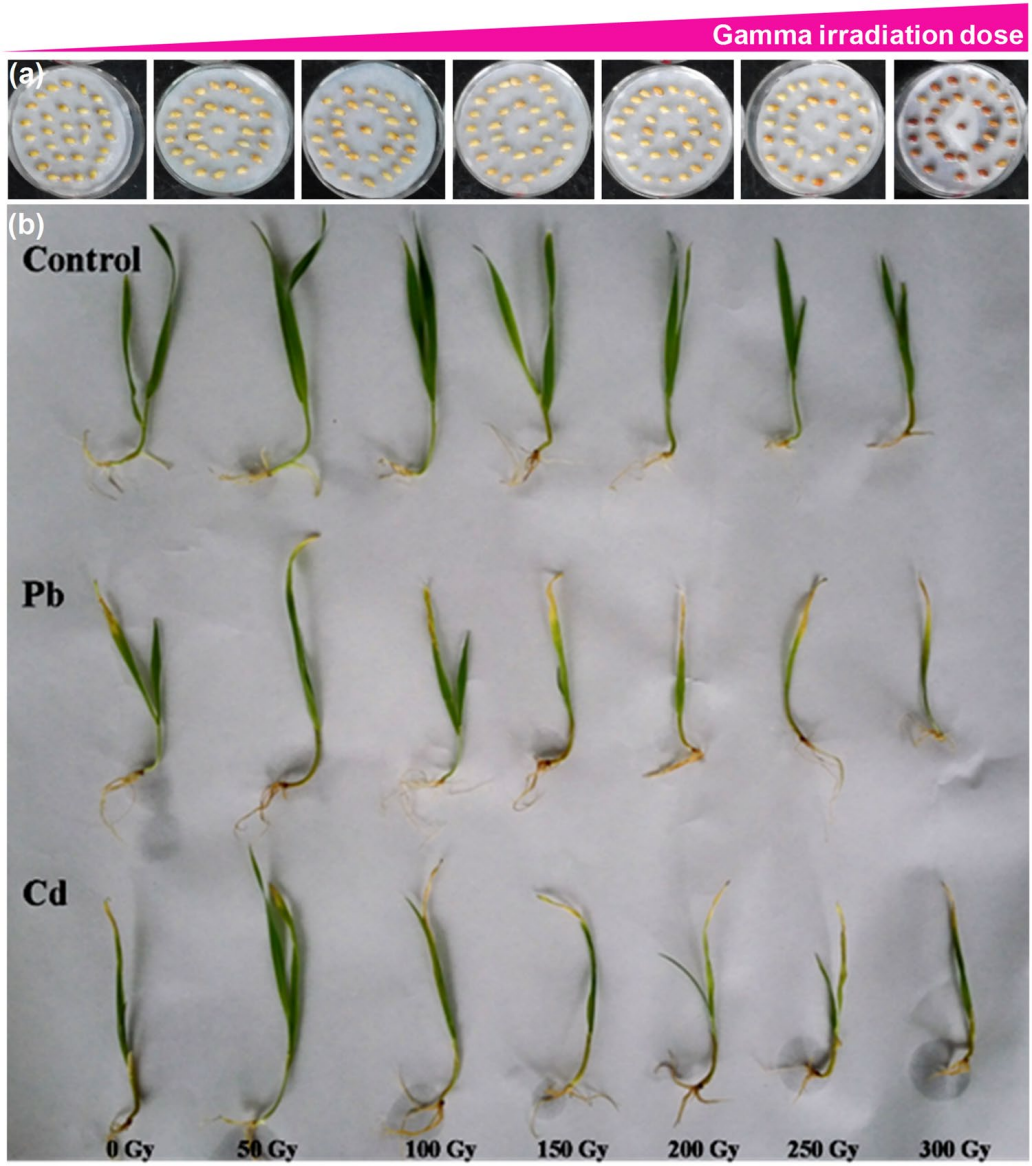
(**a**) Highland barley seeds irradiated with 0, 50, 100, 150, 200, 250 and 300 Gy gamma irradiation; (**b**) The phenotypic appearance of non-irradiated (0- Gy) and irradiated (50–300 Gy) highland barley seedlings with or without Pb/Cd treatment. Control, Pb, Cd in non-irradiated (0- Gy) and irradiated (50–300 Gy) groups correspond to non-irradiated or irradiated highland barley seedlings without heavy metal treatment, with 500 µM Pb(NO_3_)_2_ treatment and 75 µM CdCl_2_ treatment, respectively.

Excessive heavy metal accumulation can cause toxicity in plants, leading to stunted growth, chlorosis, wilting and leaf rolling^32^. Thus, the phenotypic appearance of highland barley was observed to evaluate the effect of low-dose gamma irradiation on alleviating heavy metal toxicity. Figure 2a showed highland barley seeds irradiated with 0, 50, 100, 150, 200, 250 and 300-Gy gamma irradiation, respectively. After 7-day cultivation and 2-day heavy metal stress, highland barley seedlings germinated from seeds were shown in Fig. 2b, for non-irradiated and irradiated (50–300 Gy) seedlings in control group, the highland barley leaves all presented normal phenotype. While, in Pb and Cd group, the highland barley leaves of non-irradiated and irradiated seedlings all exhibited visible wounding phenotypes, including stunted growth, chlorosis, wilting and leaf rolling. However, it was worth mentioning that 50-Gy gamma-irradiated seedlings showed improved phenotypic appearances relative to non-irradiated samples under Pb/Cd stress, with a marked reduction in the extent of chlorosis, wilting and leaf rolling of seedlings. Whereas, for other gamma irradiation doses (100–300 Gy), the injury levels of highland barley leaves were all higher than those observed in the non-irradiated sample under Pb/Cd stress. These results indicated that among the different doses of gamma irradiation, only 50-Gy gamma irradiation pretreatment could effectively alleviate the Pb/Cd-induced toxic symptoms in highland barley seedlings.

**Figure 3:**
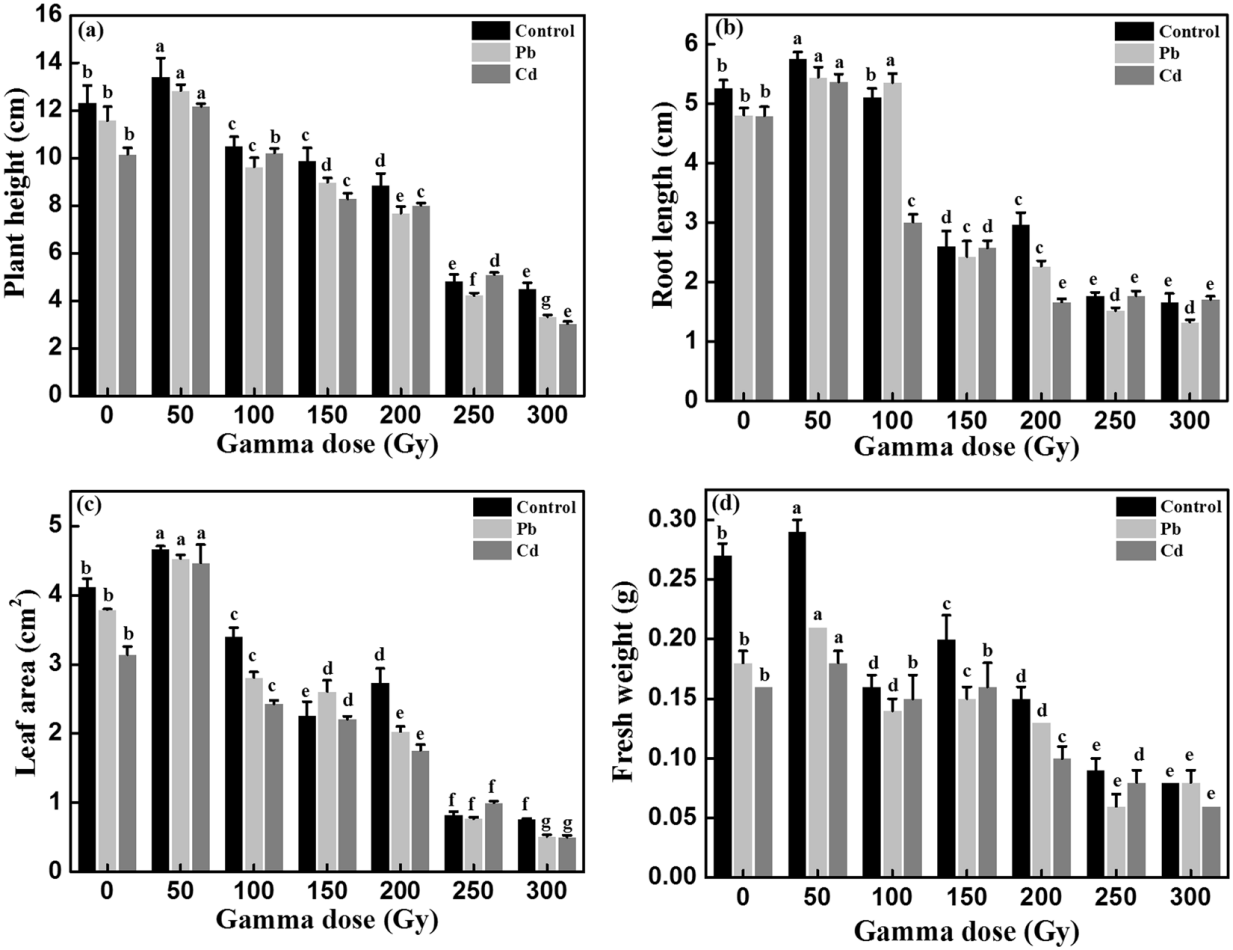
The values of plant height (**a**), root length (**b**), leaf area (**c**) and fresh weight (**d**) in non-irradiated (0- Gy) and irradiated (50–300 Gy) highland barley seedlings with or without Pb/Cd treatment. Control, Pb, Cd in non-irradiation (0- Gy) and irradiation (50–300 Gy) groups correspond to non-irradiated or irradiated highland barley seedlings without heavy metal treatment, with 500 µM Pb(NO_3_)_2_ treatment and 75 µM CdCl_2_ treatment, respectively. Vertical bars represent the mean ± SD of three independent replications (n = 3). Different letters indicate significant differences among the different doses of gamma irradiation treatments in control, Pb and Cd group at *p* < 0.05, according to LSD test.

Several plant growth parameters, including plant height, root length, leaf area and fresh weight were determined to estimate the effects of different dose gamma irradiation on highland barley growth. As shown in Fig. 3, for control group, 50-Gy gamma irradiation significantly enhanced plant height, root length, leaf area and fresh weight by 9%, 9%, 11% and 11% respectively (*p* < 0.05), compared to the non-irradiated samples. 100-Gy gamma irradiation markedly decreased plant height, leaf area and fresh weight (*p* < 0.05) and had no effects on the root length compared with the non-irradiated samples. The other gamma irradiation doses (150, 200, 250 and 300-Gy) had negative effects on highland barley growth. Under Pb stress, the values of plant height, root length, leaf area and fresh weight reached the peak at 50- Gy (11%, 13%, 19% and 12% higher than that of non-irradiated samples, respectively, *p* < 0.05) and then decreased at higher gamma irradiation doses. Moreover, relative to non-irradiated seedlings, except for 100-Gy gamma-irradiated samples with an increase of 11% in root length, the high-dose gamma irradiation treatment (100–300 Gy) all had negative effects on the plant growth. For Cd group, the change pattern of growth parameters was similar to that of Pb group. 50-Gy gamma irradiation had the maximum beneficial effects on the highland barley growth. The plant height, root length, leaf area and fresh weight were remarkably increased by 20%, 12%, 43% and 16% respectively (*p* < 0.05) compared to the non-irradiated samples. These results indicated that pretreatment with 50-Gy gamma irradiation could significantly alleviate the Pb/Cd-induced toxicity in highland barley seedlings and obviously stimulate plant growth under Pb/Cd stress.

### Effect of gamma irradiation on H_2_O_2_ and MDA content in highland barley under Pb/Cd stress

**Figure 4:**
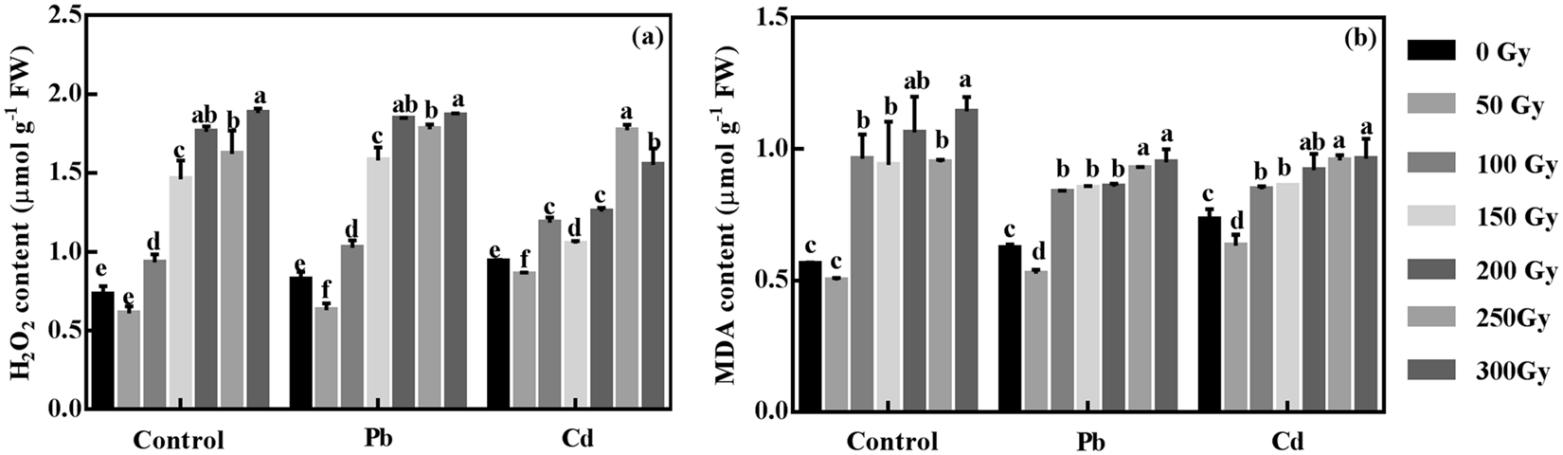
The values of H_2_O_2_ concentration (**a**) and MDA content (**b**) in non-irradiated (0- Gy) and irradiated (50–300 Gy) highland barley seedlings with or without Pb/Cd treatment. Control, Pb, Cd in non-irradiation (0- Gy) and irradiation (50–300 Gy) groups correspond to non-irradiated or irradiated highland barley seedlings without heavy metal treatment, with 500 µM Pb(NO_3_)_2_ treatment and 75 µM CdCl_2_ treatment, respectively. Vertical bars represent the mean ± SD of three independent replications (n = 3). Different letters indicate significant differences among the different doses of gamma irradiation treatments in control, Pb and Cd group at *p* < 0.05, according to LSD test.

Heavy metal stress can stimulate the production of ROS in cells, thereby leading to oxidative stress. H_2_O_2_ and lipid peroxidation product, MDA, are commonly considered as major indicators of oxidative stress^33^. To verify whether gamma irradiation could mitigate the oxidative stress induced by heavy metal in highland barley seedlings, H_2_O_2_ and MDA contents were measured. As shown in Fig. 4a, in control group, although the H_2_O_2_ level of 50-Gy irradiated seedlings was lower than that of non-irradiated sample, there was no significant difference between them. Except for 250-Gy irradiated seedlings, the H_2_O_2_ level was increased with the gamma irradiation dose and peaked at 300-Gy. Under Pb stress, 50-Gy gamma irradiation resulted in a marked decrease of 24% (*p* < 0.05) in the H_2_O_2_ level in comparison with non-irradiated samples. A distinct increase in H_2_O_2_ levels by 24%, 91%, 123%, 115% and 126% (*p* < 0.05) were observed in 100, 150, 200, 250 and 300-Gy irradiated seedlings, respectively, compared with non-irradiated samples. Moreover, similar results were also obtained in Cd group.

With respect to MDA level (Fig. 4b), there was no significant difference between non-irradiated and 50-Gy irradiated seedlings in control group. Moreover, 100, 150, 200, 250 and 300-Gy gamma irradiation resulted in a significant increase of 71%, 68%, 89%, 69% and 103% (*p* < 0.05) in MDA level, respectively, when compared with the non-irradiated samples in control group. In Pb group, 50-Gy irradiated seedlings caused a drastic decrease of 16% (*p* < 0.05) relative to the non-irradiated samples. For higher gamma irradiation doses (100–300 Gy), the MDA level was increased with the gamma irradiation dose, which resulted in a significant increase of 34%, 37%, 39%, 50% and 53% (*p* < 0.05), respectively, compared with non-irradiated samples. For Cd group, the change pattern of MDA level was similar with that under Pb stress. Based on these results, it can be concluded that pre-treatment with 50-Gy gamma irradiation could effectively alleviate Pb/Cd-induced oxidative stress.

### Effect of gamma irradiation on antioxidant enzyme activity in highland barley under Pb/Cd stress

**Figure 5:**
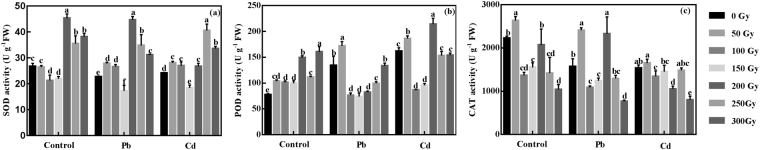
The activities of SOD (**a**), CAT (**b**) and POD (**c**) in non-irradiated (0- Gy) and irradiated (50–300 Gy) highland barley seedlings with or without Pb/Cd treatment. Control, Pb, Cd in non-irradiated (0- Gy) and irradiated (50–300 Gy) groups correspond to non-irradiated or irradiated highland barley seedlings without heavy metal treatment, with 500 µM Pb(NO_3_)_2_ treatment and 75 µM CdCl_2_ treatment, respectively. Vertical bars represent the mean ± SD of three independent replications (n = 3). Different letters indicate significant differences among the different doses of gamma irradiation treatments in control, Pb and Cd group at *p* < 0.05, according to the LSD test.

Furthermore, to understand the role of gamma irradiation in alleviating Pb/Cd-induced oxidative stress, the activities of several representative antioxidant enzymes (SOD, CAT and POD) in highland barley seedlings after different treatments were investigated. As shown in Fig. 5a, in control group, there was no significant difference in SOD activity between non-irradiated and 50-Gy irradiated seedlings. 100 and 150-Gy gamma irradiation caused a dramatic reduction of 20% and 18% (*p* < 0.05) in SOD activity compared with non-irradiated samples. While, 200, 250 and 300-Gy gamma irradiation led to a significant increase of 69%, 33% and 43% (*p* < 0.05) compared with non-irradiated samples. In Pb group, SOD activity was enhanced in all gamma irradiation doses except for 150 Gy, which resulted in a marked decline of 24% (*p* < 0.05) relative to non-irradiated samples. There was a remarkable increase of 22%, 17%, 96%, 53% and 37% (*p* < 0.05) in 50, 100, 200, 250 and 300-Gy irradiated seedlings relative to non-irradiated samples. Similarly, in Cd group, a marked decrease of 24% (*p* < 0.05) as well as significant increase of 16%, 11%, 10%, 68% and 39% (*p* < 0.05) in the SOD activity was observed in 150, 50, 100, 200, 250 and 300-Gy irradiated seedlings, respectively, compared with non-irradiated seedlings.

In the case of POD activity (Fig. 5b), 50, 100, 150, 200, 250 and 300- Gy gamma irradiation all increased POD activity in seedlings, which was 34%, 31%, 29%, 92%, 44% and 107% (*p* < 0.05) higher than that of non-irradiated samples in control group. In Pb group, POD activity of 50-Gy irradiated seedlings significantly was increased by 28% (*p* < 0.05) compared with non-irradiated samples. There was no significant difference in POD activity between non-irradiated and 300-Gy irradiated seedlings. A dramatic reduction of 43%, 45%, 39% and 26% (*p* < 0.05) in POD activity was observed in seedlings after 100, 150, 200 and 250-Gy gamma irradiation treatment, respectively, when compared with non-irradiated samples. In Cd group, compared with non-irradiated samples, the POD activity was increased by 15% and 32% (*p* < 0.05) in 50 and 200-Gy irradiated seedlings, respectively. While, the POD activity was decreased by 46% and 41% (*p* < 0.05) after 100 and 150-Gy gamma irradiation treatment. 250, 300-Gy gamma irradiation had no effects on POD activity relative to non-irradiated samples.

Additionally, in Fig. 5c, pre-treatment with 50-Gy gamma irradiation significantly increased the CAT activity, which was 18% (*p* < 0.05) higher than that of non-irradiated samples in control group. Nevertheless, 100, 150, 200, 250 and 300-Gy gamma irradiation all decreased the CAT activity to a certain extent compared with non-irradiated samples. Under Pb stress, CAT activity in 50 and 200-Gy irradiated seedling was significantly increased by 53% and 48% (*p* < 0.05) compared with non-irradiated samples. While, the other doses of gamma irradiation all led to a marked decline in the CAT activity compared with the non-irradiated samples. Under Cd stress, 50-Gy gamma irradiation slightly increased the CAT activity (7%) in seedlings compared with the non-irradiated samples, without any significant difference. The CAT activity in the other doses of gamma irradiation was all lower than that of non-irradiated samples.

### Effect of gamma irradiation on proline accumulation in highland barley under Pb/Cd stress

**Figure 6:**
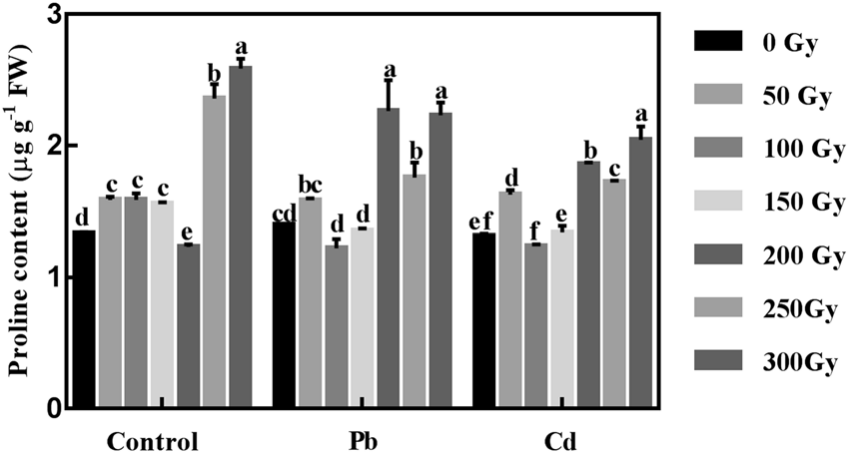
The values of proline content in non-irradiated (0- Gy) and irradiated (50–300 Gy) highland barley seedlings with or without Pb/Cd treatment. Control, Pb, Cd in non-irradiated (0- Gy) and irradiated (50–300 Gy) groups correspond to non-irradiated or irradiated highland barley seedlings without heavy metal treatment, with 500 µM Pb(NO_3_)_2_ treatment and 75 µM CdCl_2_ treatment, respectively. Vertical bars represent the mean ± SD of three independent replications (n = 3). Different letters indicate significant differences among the different doses of gamma irradiation treatments in control, Pb and Cd group at *p* < 0.05, according to the LSD test.

To further explore whether the observed low-dose-gamma-irradiation-enhanced heavy metal tolerance is related to proline accumulation in highland barley seedlings, proline levels were measured in seedlings after different irradiation and heavy metal treatments. As shown in Fig. 6, proline contents were enhanced in all irradiated seedlings except for 200- Gy irradiated samples in control group. The highest value of proline contents was recorded at 300-Gy gamma irradiation, with a distinct increase of 94% (*p* < 0.05) compared with non-irradiated samples in control group. Under Pb stress, a dramatic increase of 13%, 62%, 26% and 60% (*p* < 0.05) in proline contents was observed after 50, 200, 250, 300-Gy gamma irradiation treatment compared with non-irradiated samples. While, 100 and 150-Gy gamma irradiation led to a decrease in the proline contents relative to non-irradiated samples, without any significant difference. Moreover, the results of proline contents under Cd stress were in accordance with that under Pb stress. Proline contents in 50, 200, 250 and 300-Gy irradiated seedlings were significantly increased by 24%, 41%, 31% and 56% (*p* < 0.05) compared with non-irradiated samples in Cd group. 100 and 150-Gy gamma irradiation had no effects on the proline contents relative to non-irradiated samples. These results indicated that 50-Gy gamma irradiation or higher doses gamma irradiation (200, 250 and 300-Gy) could lead to proline accumulation under Pb/Cd stress.

### Effect of low-dose gamma irradiation on chloroplast ultrastructure in highland barley under Pb/Cd stress

**Figure 7:**
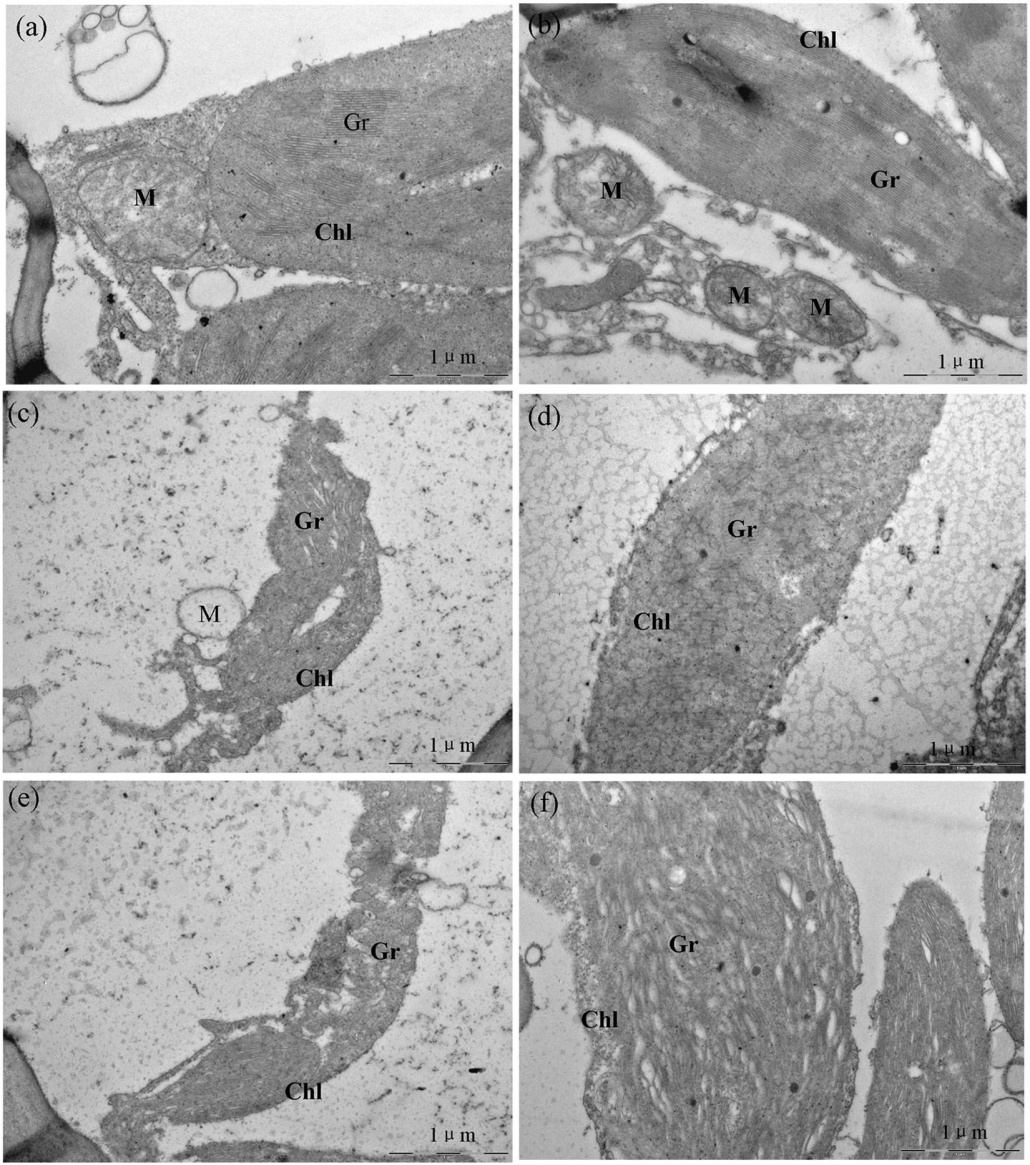
TEM micrographs of chloroplast structure in non-irradiated (0-Gy) and irradiated (50- Gy) highland barley leaves with or without Pb/Cd treatment at a magnification of ×30000: (**a**) non-irradiated highland barley without heavy metal treatment; (**b**) irradiated highland barley without heavy metal treatment; (**c**) non-irradiated highland barley with 500 µM Pb(NO_3_)_2_ treatment; (**d**) irradiated highland barley with 500 µM Pb(NO_3_)_2_ treatment; (**e**) non-irradiated highland barley with 75 µM CdCl_2_ treatment; (**f**) irradiated highland barley with 75 µM CdCl_2_ treatment. (Chl – chloroplast, Gr – granum and M – mitochondrion).

The ultrastructural changes of chloroplast morphology in highland barley after different treatments are shown in Fig. 7. Without Pb/Cd treatment, no significant change in chloroplast of non-irradiated and 50-Gy irradiated samples was observed. They all showed a typical structure, with ellipsoidal morphology, and well-organized thylakoid membranes of distinct grana and stroma regions (Fig. 7a,b). In contrast, under Pb/Cd stress, the chloroplasts of the non-irradiated plants showed damaged ultrastructures, with wavy grana and stroma thylakoids, enlarged intrathylakoidal spaces and severely deformed envelope membrane (Fig. 7c,e). In contrast, as shown in Fig. 7d,f, althought Pb/Cd treatment could also destroy the chloroplast ultrastructure in 50-Gy irradiated samples, the changes in 50-Gy irradiated plants under Pb/Cd stress were not as drastic as those observed in Pb/Cd stressed plants without gamma irradiation. Therefore, pre-treatment with 50-Gy gamma irradiation could also protect the chloroplast ultrastructure of highland barley seedlings from heavy metal destruction.

### Effect of low-dose gamma irradiation on gene expression related to heavy metal resistance in highland barley under Pb/Cd stress

**Figure 8:**
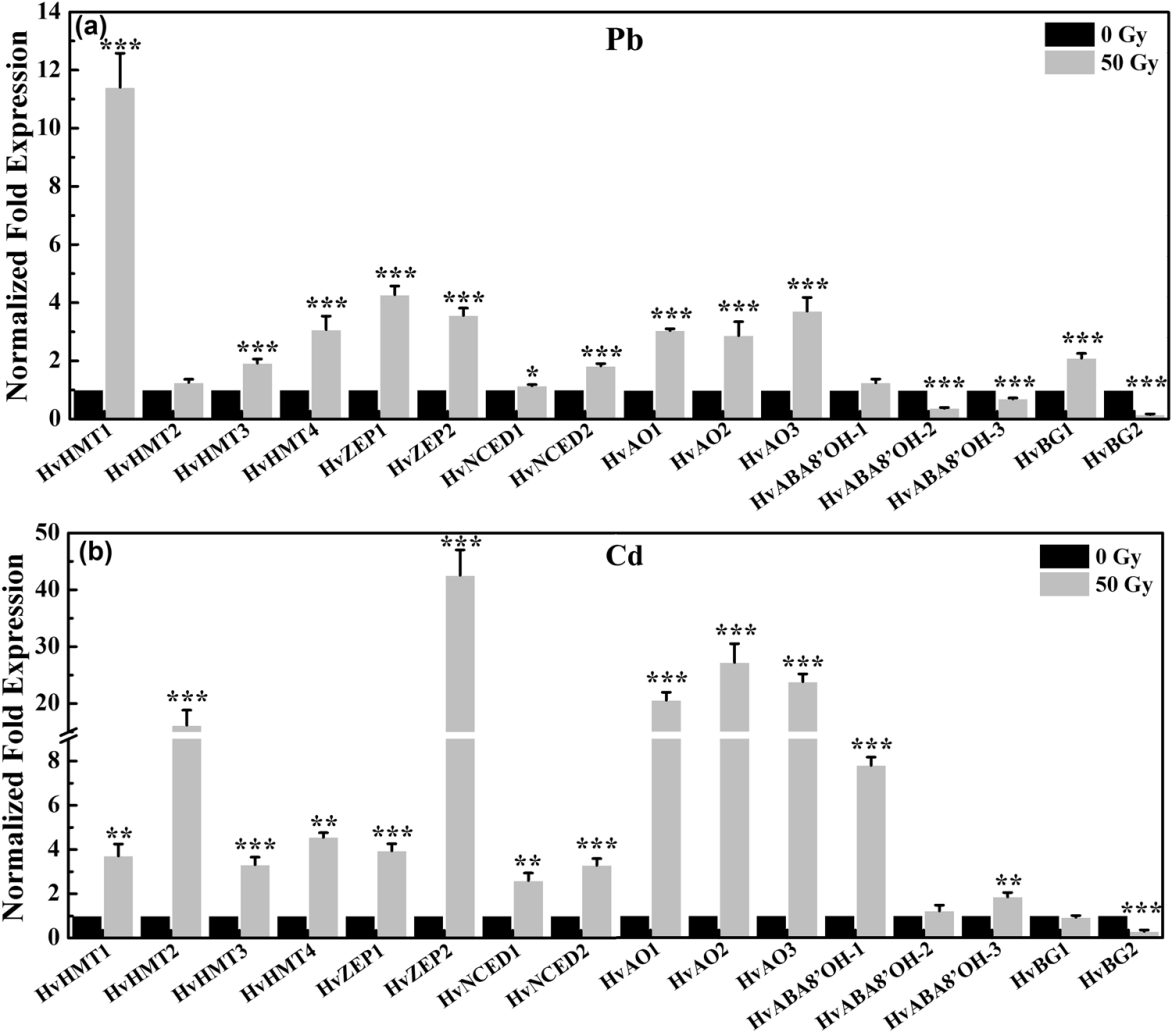
Quantitative real-time PCR analysis of genes involved in heavy metal transport (*HvHMT1*, *HvHMT2*, *HvHMT3* and *HvHMT4*), ABA biosynthesis (*HvZEP1*, *HvZEP2*, *HvNCED1*, *HvNCED2*, *HvAO1*, *HvAO2* and *HvAO3*) and ABA catabolism (*HvABA8*′*OH-1*, *HvABA8*′*OH-2*, *HvABA8*′*OH-3*, *HvBG1* and *HvBG2*) in non-irradiated (0- Gy) and irradiated (50- Gy) highland barley seedlings with 500 µM Pb(NO_3_)_2_ (**a**) and 75 µM CdCl_2_ (**b**) treatment. The results are reported as the relative expression of each gene transcripts with respect to internal standard. The relative value for the expression of each gene in non-irradiated sample was set as 1. Vertical bars represent the mean ± SD of three independent replications (n = 3). Significant difference between non-irradiated and irradiated sample for each gene is indicated with * at *p* < 0.05, ** at *p* < 0.01 and *** at *p* < 0.001, according to paired-sample t-test.

To gain insights into the molecular mechanisms underlying the enhanced heavy metal tolerance by low-dose gamma irradiation in highland barley seedlings, the expression levels of sixteen genes directly or indirectly related to heavy metal detoxification were analyzed. These sixteen genes can be divided into three groups: *HvHMT1*, *HvHMT2*, *HvHMT3* and *HvHMT4* encoding heavy metal ions transporters^18^; *HvZEP1*, *HvZEP2*, *HvNCED1*, *HvNCED2*, *HvAO1*, *HvAO2* and *HvAO3* encoding enzymes required for ABA synthesis; *HvABA8*′*OH-1*, *HvABA8*′*OH-2*, *HvABA8*′*OH-3*, *HvBG1* and *HvBG2* encoding enzymes required for ABA catabolism^20^. As shown in Fig. 8, the transcript levels of these heavy metal transport genes were all markedly up-regulated in seedlings pre-treated with 50-Gy gamma irradiation under Pb/Cd stress compared with non-irradiated seedlings, except for *HvZEP2* in Pb-stressed seedlings. The gene expression patterns of these four genes were also significantly different between Pb-stressed and Cd-stressed seedlings. Pb and Cd treatment respectively resulted in an 11.41, 1.26, 1.92 and 3.07-fold increase as well as a 3.72, 16.22, 3.31, and 4.56-fold increase in the transcript levels of *HvHMT1*, *HvHMT2*, *HvHMT3* and *HvHMT4* in 50-Gy irradiated seedlings compared with non-irradiated seedlings. Among these four genes, *HvHMT1* contributed most to the Pb metal resistance in highland barley seedlings pre-treated by 50-Gy gamma irradiation. While, *HvHMT2* contributed most to the Cd metal resistance in 50-Gy irradiated seedlings. Moreover, the expression of these seven genes related to ABA biosynthesis were all significantly enhanced in 50-Gy irradiated seedlings under Pb and Cd stress compared with non-irradiated samples. Meanwhile, compared with Pb treatment, Cd treatment presented higher transcript levels of these genes in 50-Gy irradiated seedlings, shown by a 3.94, 42.57, 2.60, 3.29, 20.57, 27.22 and 23.83-fold increase in the transcript level of *HvZEP1*, *HvZEP2*, *HvNCED1*, *HvNCED2*, *HvAO1*, *HvAO2* and *HvAO3*, respectively, relative to non-irradiated samples. Additionally, with respect to the five ABA catabolic genes, 50-Gy gamma irradiation almost had no effects on the transcript level of *HvABA8*′*OH-1* gene under Pb stress. The gene expression of *HvABA8*′*OH-2*, *HvABA8*′*OH-3* and *HvBG2* was significantly down-regulated in 50-Gy irradiated seedlings compared with non-irradiated samples under Pb stress. While, *HvBG2* gene expression was significantly enhanced in Pb-stress seedlings by 50-Gy gamma irradiation compared with non-irradiated samples. For Cd treatment, the gene expression of *HvABA8*′*OH-2* and *HvBG1* were relatively unaffected by 50-Gy gamma irradiation. Moreover, 50-Gy gamma irradiation led to a dramatic decrease in *HvBG2* gene expression, meanwhile a marked increase in *HvABA8*′*OH-1* and *HvABA8*′*OH-3* gene expression compared with non-irradiated samples under Cd stress.

## Discussions

Highland barley is one of the most economical and nutritious foods in the Tibetan Plateau of northwestern China^12–14^. In the past few decades, with the rapid development of industrialization and metal mining activities in Tibet, heavy metal has been reported to cause negative effects on soil and water quality of Tibet^7–11^. Thus, highland barley is under the potential threat of heavy metal pollution. Among the various heavy metals, Pb and Cd are considered non-essential and highly toxic, adversely affecting plant growth, development and quality, consequently leading to reduced yield^16,17^. Moreover, Pb and Cd are also readily accumulated in the aboveground parts of plants, thereby entering food chains and threatening human health^15^. Hence, improving highland barley resistance to heavy metal stress has become an urgent task to ensure sufficient food supply and food safety. It is generally accepted that low doses of gamma rays stimulate cell division growth, and development of various organisms, while high-dose gamma irradiations cause inhibitory effects. This phenomenon has been named radiation hormesis and has received considerable attention^27^. Recently, many researchers have reported that low-dose gamma irradiation can also enhance plant resistance to various abiotic stresses^31,34^. Thus, this study was performed to evaluate the effects of gamma irradiation on highland barley tolerance to heavy metal stress and unraveling its mechanisms from physio-biochemical and molecular aspects.

Growth data indicated that gamma irradiation differentially affects the plant height, root length, leaf area and fresh weight of highland barley in a dose-dependent manner in control, Pb and Cd group. 50-Gy gamma irradiation resulted in the maximal positive effects on all of the growth parameters analyzed, consistently with our initial findings (Figs 2b and 3). Moreover, the hormetic effects induced by gamma irradiation have been reported by many researchers in various plants, such as lettuce (*Lactuca sativa* var. *capitata*)^35^, chickpea (*Cicer arietum* L.)^36^, rice (*Oryza sativa* L.)^37^, black gram (*Vigna mungo* L.)^38^, okra (*Hibiscus esculentus* L.)^39^, maize (*Zea mays* L.)^40^, soybean (*Glycine max* L.)^28^, *Terminalia arjuna* Roxb. (*T. arjuna*)^41^ and *Lathyrus chrysanthus* Boiss. (*L. chrysanthus*)^42^. Although there are still no conclusive explanations for the stimulatory effects of low-dose gamma radiation, several hypotheses have been proposed. Some researchers assumed that low-dose gamma irradiation can activate RNA and protein synthesis during the early stage of germination^43^. Another hypothesis with regards to gamma stimulation is reported to be the acceleration of cell division as well as the direct or indirect activation of auxin-responsive genes^44^. Moreover, Kim *et al*. speculated that low-dose irradiation can induce the growth stimulation by changing the hormonal signaling network in plant cells or by increasing the antioxidative capacity of the cells to easily overcome daily stress factors in the growth condition^45^. Furthermore, Fan *et al*. indicated that the free radicals generated by gamma irritation in plants can act as stress signals promoting the synthesis of phenolic compounds with high antioxidant properties^46^. In contrast, seeds irradiated with high doses of gamma rays disrupted the synthesis of proteins, photosynthesis, hormone balance, leaf-gas exchange, water exchange and enzyme activity, ultimately leading to the disorders of plant physiology and morphology as well as inhibition of plant growth and development^47^.

According to the results of plant phenotypes, Pb/Cd treatment caused toxicity in highland barley, leading to stunted growth, chlorosis, wilting and leaf rolling (Fig. 2b). Similar results were observed in Mostofa’s work, reporting that excessive Cd exposure resulted in growth inhibition and biomass reduction of rice, which is correlated with the increased uptake of Cd and depletion of the photosynthetic pigments, leaf water contents, essential minerals, water-soluble proteins, and enzymatic and non-enzymatic antioxidants as well as Cd-induced oxidative stress^32^. Whereas, 50-Gy gamma irradiation could improve overall growth and biomass of highland barley after Pb/Cd treatment via enhancing the highland barley resistance to Pb/Cd stress. Our previous results suggested that low-dose gamma irradiation enhanced Pb/Cd tolerance in *Arabidopsis* seedlings, probably by modulating the physiological responses and gene expression levels related to heavy metal detoxification^31^. Moreover, Abo-Hamad *et al*. reported that the biopositive effects of low-dose gamma irradiation on plant growth under Pb/Cd stress may be attributed to the increased synthesis of phytochelatin or secondary metabolites associated with stress tolerance during seedling growth after seed irradiation^30^.

Various abiotic stresses, such as salt, drought, heat, or heavy metal stress, can trigger the generation of ROS including hydroxyl radical (•OH), singlet oxygen (^1^O_2_), superoxide anion (•O_2_^−^) and H_2_O_2_, which can lead to the unspecific oxidation of proteins, membrane lipids and DNA, thereby accompanied by lipid peroxidation, H_2_O_2_ accumulation and an oxidative burst in the cell^48^. In this study, the highland barley seedlings treated with Pb/Cd exhibited severe oxidative stress in leaf tissues as shown by markedly increased levels of H_2_O_2_ and MDA compared to seedlings without heavy metal exposure. 50-Gy gamma irradiation can effectively reduce the Pb/Cd-induced oxidative stress as indicated by lower levels of H_2_O_2_ and MDA in irradiated seedlings than those in non-irradiated seedlings in response to Pb/Cd stress (Fig. 4), consistently with our previous results in *Arabidopsis* seedlings^31^. As a response to ROS overproduction, plant cells activate endogenous enzymatic defense mechanisms against oxidative stress^49^. Three important antioxidant enzymes of SOD, CAT and POD were investigated in this study, which form the first and most important line of antioxidant defense^33^. SOD catalyzes the dismutation of •O_2_^−^ to H_2_O_2_ and O_2_, then both CAT and POD can decompose H_2_O_2_ into H_2_O and O_2_^49^. Moreover, the activities of SOD, CAT and POD were all significantly increased by 50-Gy gamma irradiation in response to Pb/Cd stress or under non-stress condition (Fig. 5), contributing most to the lower H_2_O_2_ and MDA content in irradiated seedlings (Fig. 4). A similar trend was also observed in the irradiated *Arabidopsis* seedlings under Pb/Cd stress^31^. Actually, it has been reported that the activities of scavenging enzymes, such as POD, CAT, SOD, and ascorbate peroxidase (APX), are generally increased in various plant species by the treatment of ionizing radiation^50^. Moreover, it is generally accepted that ionizing radiation can trigger the production of ROS by interacting with atoms or molecules in the cell, which is called water radiolysis^51^. Thus, it is possible that low-dose gamma irradiation can stimulate SOD, CAT and POD activity to enhance the basal antioxidant capacity to overcome oxidative stress induced by water radiolysis. Taken together, our results suggest that the increased activities of SOD, POD, and CAT induced by 50-Gy gamma irradiation can scavenge the excess ROS produced by Cd/Pb stress, consequently leading to an enhanced heavy metal tolerance in highland barley seedlings.

Another important mechanism in regulating heavy metal-induced toxicity is associated with the accumulation of proline. As a cytosolic osmoticum, proline can be accumulated in a wide range of organisms to protect cells against various environmental stresses by balancing the osmotic strength of the cytosol with that of the vacuole and the external environment^52^. Meanwhile, proline can also scavenge •OH as a non-enzymatic antioxidant, thereby stabilizing the structure and function of macromolecules such as DNA, proteins, and lipids^53^. The present study demonstrated that the proline levels significantly increased in the irradiated highland barley seedlings under Pb/Cd stress compared with the non-irradiated samples (Fig. 6), which is in agreement with our previous results of *Arabidopsis* seedlings under Pb/Cd stress^31^. Moreover, Abo-Hamad *et al*. reported that *B. rapa* (L.) plants irradiated with low-dose gamma rays promoted the accumulation of proline under Cd stress^54^. Given that plant-water imbalance and oxidative stress are general consequences of heavy metal toxicity, our results suggest that the observed enhanced heavy metal tolerance in irradiated highland barley seedlings was partly due to the proline accumulation, which can not only improve water imbalance in plants as an osmolyte, but also alleviate oxidative stress as an antioxidant. In addition, in control group, different doses of gamma irradiation also promoted proline accumulation, which is in good agreement with the findings of Akshatha *et al*. who observed that the proline content in *T. arjuna* increased with the increasing gamma irradiation dose, indicating that proline accumulation is an important protective mechanism in *T. arjuna* for better tolerance of plants to radiation-induced stress^41^. Thus, 50-Gy gamma irradiation could also cause stress to highland barley seedlings, consequently resulting in proline accumulation.

Chloroplast, as the site of chlorophyll formation and photosynthesis, is important for plant growth^55^. In this study, Pb/Cd stress severely destroyed the chloroplast ultrastucture with swelling of thylakoids, degradation of internal chloroplast membranes and leaving the integrity of chloroplast envelopes based on the TEM observations (Fig. 7c,e). Similar findings have also been reported by Hou *et al*. in duckweed^56^. Excessive heavy metal could not only cause oxidative damage to chloroplast membranes and thylakoids via increased production of ROS^16^, but also directly destroy the structure and function of chloroplasts by binding with-SH group of enzyme^57^. While, the low-dose gamma irradiation significantly alleviated the ultra-structural disorders caused by Pb/Cd stress in highland barley seedlings (Fig. 7d,f). Moreover, Moussa has reported that low-dose gamma irradiation (20-Gy) could also enhance drought tolerance in soybean via increasing the chloroplast size reduce by drought stress, rebuilding the chloroplast structure to some extent and increasing chlorophyll content^28^. Thus, our results indicate that the protective effects on chloroplast ultrastructure caused by low-dose gamma irradiation, probably due to reducing the Pb/Cd-induced oxidative damage on chloroplasts such as peroxidation of chloroplast membranes via increase of antioxidant enzyme activities and accumulation of proline, ultimately result in an improved photosynthetic capacity, which may also play an important role in the heavy metal tolerance induced by low-dose gamma irradiation in highland barley seedlings.

The transcriptional profiles of *Arabidopsis* genes in response to gamma irradiation revealed that most of genes related to general metabolism, ROS scavenging and stress signaling are affected by gamma irradiation^58^. However, there is still little information available on the transcriptional responses of highland barley seedlings pre-treated with low-dose gamma irradiation to Pb/Cd stress. In the current study, four heavy metal transport genes were markedly up-regulated by 50-Gy gamma irradiation under Pb/Cd stress (Fig. 8), indicating that these genes are all involved in the heavy metal tolerance induced by 50-Gy gamma irradiation in highland barley seedlings. It is generally accepted that plant hormone ABA signalling is essential for the modulation of a variety of physiological processes, including seed dormancy, stomata closure, senescence, and adaptive responses to stresses (such as cold, drought, heavy metal and salinity)^19^. A recent report on the transcriptome profiles of growth-promoted rice seedlings treated with a low-energy ion beam revealed that the expression of genes related to ABA synthesis and signal transduction was obviously up-regulated, implicating the possible role of ABA signalling in the regulation of the encouraged stress^59^. However, it is unclear how low-dose gamma irradiation regulates the expression of genes responsible for the ABA homeostasis in highland barley seedlings. In this study, the transcription levels of genes encoding enzymes required for ABA synthesis and catabolism with and without 50-Gy gamma irradiation under Pb/Cd stress were analyzed (Fig. 8). These data showed that most of the ABA biosynthesis-related genes selected were highly up-regulated in 50-Gy irradiated samples compared with the non-irradiated samples. Similar results were observed in Qi’s work, reporting that low-dose gamma irradiation triggers ABA accumulation in *Arabidopsis* ^59^. Thus, based on the vastly different transcriptional profiles of genes regulating ABA metabolism in 50-Gy irradiated seedlings under Pb/Cd stress, it is hypothesized that the increase in transcript levels of genes associated with ABA biosynthesis and decrease in transcript levels of ABA catabolic genes may be contributed to elevating ABA levels in highland barley, consequently improving the heavy metal tolerance.

In conclusion, the results in this study show that pre-treatment with low-dose gamma irradiation (50- Gy) can effectively enhance heavy metal tolerance in highland barley seedlings by decreasing H_2_O_2_ and MDA content, meanwhile increasing the activity of antioxidant enzyme (SOD, POD and CAT), consequently alleviating the heavy metal-induced oxidative stress, as well as promoting proline accumulation, protecting chloroplasts ultrastructure from heavy metal destruction and up-regulating the expression of genes associated with heavy metal detoxification and ABA biosynthesis, meanwhile down-regulating the expression of genes related to ABA degradation. Therefore, this work extends the available knowledge of the physio-biochemical and molecular mechanisms underlying the heavy metal tolerance induced by gamma irradiation in highland barley. Furthermore, as an alternative strategy, low-dose gamma irradiation can provide a potentially feasible way for improving crop production in heavy metal contaminated soils.

## Methods

### Plant materials

Seeds of the highland barley cultivar ‘Kunlun15′ (Hordeum vulgare ssp. vulgare), kindly provided by the Qinghai Academy of Agricultural Sciences, Qinghai Province, China, were subjected to gamma irradiation treatment.

### Gamma irradiation

Gamma irradiation of highland barley seeds was performed using a ^60^Co gamma source at a dose rate of 6.25 Gy/min. The doses of exposure involved in this study were 0, 50, 100, 150, 200, 250 and 300- Gy. Seeds treated by 0-Gy gamma irradiation were used as the non-irradiated control sample.

### Growth conditions and heavy metal treatment

Immediately after gamma irradiation, both non-irradiated and irradiated (50- Gy) seeds were sanitized with 5% sodium hypochlorite for 15 min, then thoroughly washed with sterile distilled water, and germinated at 25 °C in the dark for 72 h. The germinated seeds were transferred into a plant growth chamber at 23 °C with a 16-h-light/8-h-dark cycle. For heavy metal stress, the first true leaves of highland barley seedlings were fully expanded at the first true leaf stage after 7-day growth, and then submerged in Hoagland’s solution containing 500 µM Pb(NO_3_)_2_ or 75 µM CdCl_2_ for 48 h. The retrieved seedlings were then immediately frozen in liquid nitrogen and stored at −80 °C. This study consisted of six treatment groups as follows: non-irradiated sample without heavy metal treatment (Control at 0-Gy), non-irradiated sample with 500 µM Pb(NO_3_)_2_ treatment (Pb at 0-Gy), non-irradiated sample with 75 µM CdCl_2_ treatment (Cd at 0-Gy), irradiated sample without heavy metal treatment (Control at 50-Gy), irradiated sample with 500 µM Pb(NO_3_)_2_ treatment (Pb at 50-Gy) and irradiated sample with 75 µM CdCl_2_ treatment (Cd at 50-Gy). Each treatment was replicated three times under the same experimental conditions (n = 3).

### Determination of growth parameters

Following pre-culturing for 7 days, samples were collected from 10 randomly selected plants per replicate. Root length, plant height, leaf area, and fresh weight were then simultaneously measured. Root length was determined by measuring the mean length of the longest roots. Plant height was estimated by measuring the length from the bottom of the main stem to the end of the emerging third leaf. Leaf area was calculated by using the formulae: leaf area = leaf length × maximum blade width × 0.833. To evaluate fresh weight, the seedlings were weighed after being separated from the culture medium and the roots were washed thoroughly with distilled water and then blotted with tissue paper.

### Measurement of H_2_O_2_ contents

H_2_O_2_ content was measured according to the method described by Patterson *et al*.^60^. 0.20 g of fresh highland barley leaves were extracted in 5 ml of pre-cooled acetone, followed by centrifugation at 10000 rpm/min  for 15 min at 4 °C. The supernatant was removed and reacted with a mixture of titanium tetrachloride (TiCl_4_) in 10% hydrochloric acid (HCl) (v/v). Absorption at 410 nm was recorded, and the concentration of H_2_O_2_ was determined using a standard curve plotted with known concentrations of H_2_O_2_. The H_2_O_2_ concentration was calculated relative to fresh weight.

### Analysis of lipid peroxidation

Lipid peroxidation levels in fresh highland barley leaves were calculated based on MDA content, which was measured as previously described by Shalata and Neumann^61^. 0.20 g of samples were collected and homogenized thoroughly in 10% trichloroacetic acid (TCA), followed by centrifugation for 10 min at 4000 rpm/min. Then, 2 ml of the supernatant samples were mixed with 2 ml 0.6% (w/v) TCA and incubated in boiling water for 15 min. The MDA content was monitored by measuring the absorbance at 532 and 450 nm and calculated on a fresh weight basis.

### Antioxidant enzyme activity assay

To extract enzymes, 0.20 g of fresh highland barley leaf samples were homogenized separately with 2 ml of 50 mM ice-cold sodium phosphate buffer solution (PBS, pH 7.8) containing containing 4% polyvinylpyrrolidone (PVP) and 1 mM ethylene diamine tetraacetic acid (EDTA) in pre-chilled mortars. Homogenates were centrifuged at 10000 rpm/min at 4 °C for 15 min. Supernatants containing crude enzyme were analyzed for activities of SOD, CAT and POD. All procedures were performed at 4 °C.

SOD (EC 1.15.1.1) activity was evaluated according to the method described by Giannopolitis and Ries^62^, which is based on monitoring the inhibition of the photochemical reduction of nitrobluete- trazolium (NBT). 3 ml reaction mixture containing 50 mM PBS (pH 7.8), 0.1 mM EDTA, 130 mM methionine, 0.75 mM NBT, 0.02 mM riboflavin, and 0.1 ml enzyme extract. Riboflavin was added as the last component, and the reaction mixtures were illuminated for 15 min at a light intensity of 5000 lx. Non-illuminated and illuminated reactions without the supernatant served as calibration standards. One unit of SOD activity was defined as the amount of enzyme required to cause a 50% inhibition of the reduction of NBT recorded at 560 nm. CAT (EC 1.11.1.6) activity was estimated by measuring the initial rate of H_2_O_2_ disappearance^63^. The reaction solution (3 ml) consisted of 50 mM PBS (pH7.0), 20 mM H_2_O_2_, and 0.1 ml enzyme extract. A decrease in H_2_O_2_ was monitored at 240 nm for at least 3 min. POD (EC 1.11.1.7) activity was measured by the guaiacol method^64^. The enzyme extract (0.02 ml) was added to the reaction mixture containing 0.02 ml guaiacol solution and 0.01 ml H_2_O_2_ solution in 3 ml of PBS (pH 7.0). The addition of the enzyme extract started the reaction, and the increase in absorbance was recorded at 470 nm for 5 min. The SOD, CAT and POD activities were performed as total SOD, CAT, and POD activity divided by fresh weight.

### Measurement of proline content

A colorimetric methods were used to measure the proline content of highland barley leaves^65^. First, 0.20 g of highland barley leaves were harvested and homogenized in liquid nitrogen. Tissue powders were resuspended in 3% sulphosalicylic acid and incubated in boiling water for 10 min. After centrifugation, 2 ml supernatant were mixed with 4 ml acid ninhydrin, 2 ml acetic acid and 2 ml distilled water to a final volume of 10 ml. The mixture was then incubated at 100 °C for 1 h. The reaction mixtures were then extracted with 4 ml toluene, and the upper phases were collected. Absorbance was read at 520 nm. The concentration of proline was determined from a standard curve and calculated on a fresh weight basis.

### Transmission electron microscopy detection

Leaf samples of highland barley leaves were cut into pieces of 1–1.5 cm in length and fixed overnight in 2.5% glutaraldehyde (v/v) in 0.1 M PBS (pH 7.4) at 4 °C. The samples were washed three times with the same PBS and then fixed in 1% osmium tetraoxide for 1 h, and washed for 10 minutes three times in 0.1 M PBS. Samples were dehydrated in a graded series of ethanol (50%, 60%, 70%, 80%, 90%, 95% and 100%) and then washed in absolute acetone for 30 min to achieve complete dehydration. Samples were then embedded in Spurr’s resin for 24 h. Ultra-thin sections (70 nm) were cut on an ultramicrotome (Leica EM UC7, Germany), mounted on copper grids and observed by a transmission electron microscope (JEM-1400, JOEL, Japan) at 80.0 KV.

### RNA isolation and real-time quantitative RT-PCR

Total RNA was isolated from 0.10g  of 7-day-old highland barley seedlings germinated from non-irradiated and irradiated seeds exposed to 500 μM Pb(NO_3_)_2_ or 75 μM CdCl_2_ with RNAiso TM Plus (TaKaRa, Japan). Highland barley cDNA was synthesized using M-MLV reverse transcriptase (Invitrogen, USA) according to the manufacturer’s instructions. The cDNA samples were used as templates for quantitative reverse transcriptase PCR (qRT-PCR), which was performed with a Mastercycler EP Realplex thermal cycler (Eppendorf, Germany). The final reaction mixture of 20 μl volume consisted of 10 μl of 2 × SYBR^®^ Premix *Ex Taq* ^TM^ (Takara Bio, Japan), 2 μl of the reverse transcription reaction (1:5 diluted), and 0.5 μl of each gene-specific primers. The PCR protocol used was: 94 °C for 3 min, 40 cycles of 94 °C for 30 s, 55 °C for 45 s and 72 °C for 45 s. The SYBR-specific fluorophore was utilized as a quantitative tool to monitor the reactions. Three repeats were performed for each cDNA measurement. Based on the released sequences of heavy metal transport/detoxification in barley database (GenBank ID of *HvHMT1*, *HvHMT2*, *HvHMT3*, *HvHMT4* is MLOC_18537, MLOC_34972, MLOC_62248 and MLOC_1373, respectively)^18^, the primer pairs were designed for qRT-PCR. The primer pairs of genes involved in ABA biosynthesis and catabolism were referred to Sreenivasulu^20^. Data were analyzed using the Realplex 2.2 software (Eppendorf, Germany) to calculate cycle threshold (CT) values. The data were analyzed using the comparative C_t_ (2^−ΔΔCt^) method^66^. To compare the data from different PCR runs or cDNA samples, the C_t_ values for the genes were normalized to the C_t_ value of the control. The barley actin gene (*AY145451*) was used as an internal control, which is a house keeping gene included in each PCR run. The primers for each gene were listed in Table 1.

**Table 1:** The accession number and primer sequence of each gene in the real-time quantitative RT-PCR assay.

Gene	Oligo Sequence	
	Forward	Reverse
*HvHMT1*	5′-CAGAAGAAGGTGCTCAAGAC-3′	5′-GTTATACGAGGAAGTGGACC-3′
*HvHMT2*	5′-CAAGATCAAGCAGGAGGAAT-3′	5′-GATGAGAGACCGCTTGATC-3′
*HvHMT3*	5′-GTGGAGATGCAGATGAACAT-3′	5′-CAACACCTTCTTCTGGCTC-3′
*HvHMT4*	5′-CTCAAGGACAACAAGATGAC-3′	5′-GACCTTCTTGTCGTCTTTCT-3′
*HvZEP1*	5′-GCGAGAGGCGGGGGAGAAGT-3′	5′-TGGTGACAAGGGGTGGCTGAAG-3′
*HvZEP2*	5′-CTTCCTGGCTCGTCGGTTCGTC-3′	5′-GCTGGGAGTGGAGGGCGTGTAA-3′
*HvNCED1*	5′-CCAGCACTAATCGATTCC-3′	5′-GAGAGTGGTGATGAGTAA-3′
*HvNCED2*	5′-CATGGAAAGAGGAAGTTG-3′	5′-GAAGCAAGTGTGAGCTAAC-3′
*HvAO1*	5′-TCCCTGCGCCAATGACACA-3′	5′-GTTGACGGCACCGGAATCTTG-3′
*HvAO2*	5′-TCTTGTGCGCCCCTCATCTTCA-3′	5′-TCCCTTGCCCAACCTCAACACCT-3′
*HvAO3*	5′-GTGCGAGCGCCAGGTGATGT-3′	5′-TCGGCGCTGTGGAGGTTCTTT-3′
*HvABA8*′*OH-1*	5′-AGCACGGACCGTCAAAGTC-3′	5′-TGAGAATGCCTACGTAGTG-3′
*HvABA8*′*OH-2*	5′-GAGATGCTGGTGCTCATC-3′	5′-ACGTCGTCGCTCGATCCAAC-3′
*HvABA8*′*OH-3*	5′-CCGGCGGCAGCGTCTTCT-3′	5′-GTGTTGCCGTCCTGGGTGTCC-3′
*HvBG1*	5′-CCTACGTCGCCGCCCATAACA-3′	5′-GCCCGTCTTGCAGCCAGGATA-3′
*HvBG2*	5′-GCCGGTGGGAACTCAGCAACAG-3′	5′-GTCGGCAGGTGAGTCGGTAGCA-3′
*AY145451*	5′-GACTCTGGTGATGGTGTCAGC-3′	5′-GGCTGGAAGAGGACCT CA-3′

### Statistical analysis

All data were obtained from three independent replicate experiments (n = 3). The values from all experiments were expresses as the mean ± standard deviation (SD). Statistical analysis was performed using SPSS statistical package 17.0 (SPSS Inc., USA). An analysis of variance (ANOVA) was conducted to compare the effects of gamma irradiation on highland barley growth as well as H_2_O_2_, MDA, and proline levels and antioxidant enzyme activity (SOD, CAT and POD) in highland barley under Pb/Cd stress. The significant differences among treatments were identified by least significant difference (LSD) test with a confidence level at *p* < 0.05, which is a sensitive two-step testing method for pairwise comparisons among all groups. Moreover, the paired-sample t-test was applied to evaluate the significant difference of gene expression between the non-irradiated and irradiated highland barley under Pb/Cd stress. The difference is expressed as * at *p* < 0.05, ** at *p* < 0.01, and *** at *p* < 0.001.
